# Promising Links between Meditation and Reduced (Brain) Aging: An Attempt to Bridge Some Gaps between the Alleged Fountain of Youth and the Youth of the Field

**DOI:** 10.3389/fpsyg.2017.00860

**Published:** 2017-05-30

**Authors:** Florian Kurth, Nicolas Cherbuin, Eileen Luders

**Affiliations:** ^1^Department of Psychiatry and Biobehavioral Sciences, Cousins Center for Psychoneuroimmunology, Semel Institute for Neuroscience and Human Behavior, UCLA School of MedicineLos Angeles, CA, United States; ^2^Centre for Research on Ageing Health and Wellbeing, Australian National UniversityCanberra, ACT, Australia

**Keywords:** aging, brain, meditation, mindfulness, MRI

## Abstract

Over the last decade, an increasing number of studies has reported a positive impact of meditation on cerebral aging. However, the underlying mechanisms for these seemingly brain-protecting effects are not well-understood. This may be due to the fact, at least partly, that systematic empirical meditation research has emerged only recently as a field of scientific scrutiny. Thus, on the one hand, critical questions remain largely unanswered; and on the other hand, outcomes of existing research require better integration to build a more comprehensive and holistic picture. In this article, we first review theories and mechanisms pertaining to normal (brain) aging, specifically focusing on telomeres, inflammation, stress regulation, and macroscopic brain anatomy. Then, we summarize existing research integrating the developing evidence suggesting that meditation exerts positive effects on (brain) aging, while carefully discussing possible mechanisms through which these effects may be mediated.

## Introduction

Life expectancy has been rising consistently for more than a century, and it is expected that this trend will continue (Oeppen and Vaupel, 2002). In the United States, the percentage of the population aged 65 and older is projected to grow from 13.7 to 20.9% between 2012 and 2050. Furthermore, over the same time span, the percentage of the population aged 85 and older is likely to increase from 1.9 to 4.5% (Ortman et al., 2014). On a broader geographical scale, the percentage of the population aged 60 and older is estimated to double worldwide by 2050 (WHO, 2015). While this is generally good news, old age is also a major risk factor for dementia and other neurodegenerative diseases, and it is therefore expected that the prevalence of age-related pathological conditions will rise in upcoming years. For example, over 35 million people were suffering from dementia worldwide in 2010, and this number is expected to triple by 2050 (WHO, 2012). In other words, a longer life expectancy might come at the expense of a healthy life. Thus, in the current absence of a general cure for age-related pathological conditions or disease-modifying treatments for dementia, it is important to direct more attention to the development of effective preventative approaches to brain aging and neurodegeneration.

Recent research revealed a reduced negative correlation between age and brain gray matter in long-term meditation practitioners compared to age-matched controls (Luders et al., 2015). We interpreted these findings as possibly indicating that meditators' brains are less affected by aging processes Importantly, this is not an isolated observation and, over the past decade, scientific evidence suggesting that meditation may help us to hold on longer to young(er) brains, bodies, and minds has been accumulating (Epel et al., 2009; Gard et al., 2014; Koike and Cardoso, 2014; Luders, 2014; Marciniak et al., 2014; Luders and Cherbuin, 2016). Thus, in light of a steadily increasing life expectancy, meditation could be an effective means to better maintain brain tissue, preserve cognitive, and emotional reserves, and to diminish the risk of dementia and other age-related neurodegenerative diseases. However, before implementing prevention and/or intervention strategies, systematic, rigorous studies are clearly necessary to fill existing research gaps. Moreover, findings already present seem to require a better integration to construct a solid basis on which to further develop this field. Thus, in the first part of this article (Section Effects and Mechanisms of Aging), we review and discuss relevant theories and biological mechanisms implicated in aging and likely to be modulated by meditative practice. In the second part (Section Effects and Possible Mechanisms of Meditation), we summarize and attempt to integrate the increasing evidence suggesting that meditation exerts positive effects on (brain) aging; possible mechanisms through which these effects may be mediated are discussed. Note that, throughout the text, the term “meditation” is used to refer to any type of mind-body practice (for details on different types of meditation as well as mindfulness practices, please refer to Lutz et al., 2008; Lippelt et al., 2014; Marciniak et al., 2014).

## Effects and mechanisms of aging

Aging is the result of a multitude of complex direct and indirect mechanisms. While many of these mechanisms are still not fully understood, others have already been well-characterized (for review, see Lopez-Otin et al., 2013). Given the extensive literature on this topic, a comprehensive summary of the mechanisms and effects of normal brain aging is beyond the scope of this review. Instead, here we focus on selected aging-related processes for which effects of meditation have been reported, such as related to telomeres, inflammation, stress regulation, and macroscopic brain anatomy.

### Telomeres

*In vitro*, diploid cells can only replicate a finite number of times before cellular senescence and programmed cell death occur (Hayflick and Moorhead, 1961). This limited cell life-cycle is due to the progressive shortening of telomeres—the terminal sections of the chromosomal deoxyribonucleic acid (DNA) strands in eukaryotic cells that cannot be replicated completely during mitosis (Blackburn et al., 2006, 2015). In addition to this programmed mechanism, it has been established that superoxide and other free radicals, such as reactive oxygen species (ROS), damage cellular structures, including DNA (Lopez-Otin et al., 2013). ROS are constantly produced by normal cellular metabolic processes or immune responses, and may also result from exogenous sources (e.g., radiation, chemicals, etc.). Within the cell, ROS result in oxidation and subsequent alterations of membranes, organelles, proteins, and the DNA, potentially impairing physiological functioning overall. Particularly in the DNA, such oxidations can result in devastating alterations of function. While most of the DNA is consistently restored, telomeres are covered by shelterin proteins that limit repair. Thus, it has been proposed that telomeres encode the cumulative DNA damage and with it the probability of devastating mutations (Blackburn et al., 2015). Importantly, cumulative damage of the telomeres has the same effects as progressive shortening and leads to altered gene transcription, altered cell functioning as well as mitochondrial dysfunction, and ultimately programmed cell death.

Interestingly, **telomere attrition** also increases ROS production via altered signaling and consequent mitochondrial dysfunction, which results in an increased release of ROS into the cell (Sahin et al., 2011). While an increase of ROS will initially result in increased compensatory and repair mechanisms, those mechanisms will fail above a certain threshold of ROS (Hekimi et al., 2011; Lopez-Otin et al., 2013). Thus, shortened or damaged telomeres can actively induce an environment that will lead to further telomere attrition, cellular senescence, cell cycle arrest, and ultimately apoptosis. While telomere attrition may play a protective role in avoiding mutations and DNA damage, telomeres can nevertheless be repaired and elongated by a specific enzyme, telomerase, which is enriched in a variety of stem cells (Blackburn et al., 2015). Although telomerase can help repair accumulated DNA damage and re-extend shortened telomeres, this enzyme is low (sometimes even undetectable) in many human cells, consequently resulting in unremitting telomere attrition (Blackburn et al., 2006, 2015; Lopez-Otin et al., 2013).

KEY CONCEPT 1 **Telomere attrition**

Telomeres are known to shorten with every cell division. Further, DNA damage in this region is not readily repaired, but accumulates over time. This attrition of the telomeres probably encodes the potential accumulated risk for mutations and dysfunction over the lifetime of a cell. Increasing telomere attrition results in increased repair mechanisms, and finally cell death.

### Inflammation

Aging does not only affect intra-cellular biochemical processes, but also presents with changes in cell signaling, including endocrine, neuroendocrine, and neuronal communication (Lopez-Otin et al., 2013). One of these changes manifests as a weakened immune response as well as increased inflammatory signaling and has been described as central to aging. Age-associated **inflammation** was reported to inhibit epidermal stem cell function (Doles et al., 2012) and also has been discussed as an underlying factor of diseases, such as type 2 diabetes, atherosclerosis, and obesity (Lopez-Otin et al., 2013). In other words, while inflammation is a central factor of the immune response and essential for tissue regeneration and healing, this complex response becomes less specific and less effective with age. The increased inflammatory signaling that accompanies aging in mammals has been coined “inflammaging” (Salminen et al., 2012a) and represents a smoldering pro-inflammatory phenotype. Several reports point to the pro-inflammatory transcription factor NF-κB (Nuclear Factor κB) as a central element of inflammaging (Helenius et al., 1996; Adler et al., 2007; Salminen et al., 2008; Haustead et al., 2016). Moreover, inflammaging can be fueled by increased ROS and pro-inflammatory pathways. However, cause and effect on the cellular level are deeply intertwined (Salminen et al., 2012a,b; Lopez-Otin et al., 2013) and point to a complex relationship between cellular senescence, telomeres, and inflammation: As described in Section Telomeres, increased ROS—resulting, for example, from inflammatory processes, mitochondrial dysfunction, or indirectly from telomere attrition itself—may lead to increased oxidative stress and associated activation of the pro-inflammatory NF-κB pathway. Telomere attrition itself leads to the release of shelterin proteins, among them RAP1 (Ras-proximate-1), that normally cover the telomeres. Set free, RAP1 activates the NF-κB pathway, thus further increasing a pro-inflammatory state (Ye et al., 2014).

KEY CONCEPT 2 **Inflammation**

A more pro-inflammatory state along with a less focused (i.e., weaker and less localized) response may be observed in aging, coined “inflammaging.” This state weakens the immune response and wound healing on the one hand, and increases stress on the tissue overall on the other.

### Stress regulation

Stress enables individuals to effectively respond to threatening situations arising both from their internal environment (e.g., hypotension, pain) or external environment (e.g., cold, predators), and to maintain homeostasis. One mechanism involved in the so-called “stress response” is the hypothalamus-pituitary-adrenal (HPA) axis. The HPA axis describes an endocrine signaling cascade that starts with the release of corticotrophin releasing hormone (CRH) in the hypothalamus, which causes the release of adrenocorticotropic hormone (ACTH) in the pituitary gland, that then triggers the release of **glucocorticoids** (cortisol in humans) from the adrenal glands. The activation of the HPA axis leads to wide ranging-effects, including raised blood pressure and raised heart rate, increased metabolism, and increased psychological alertness, as well as decreased inflammatory and decreased immune responses. While such modulations are essential to survival and well-being, chronic activation of the stress response leads to adverse outcomes. For example, high basal levels of glucocorticoids, if sustained over time, lead to increased ROS production, and increased risk of DNA and cellular damage (Garcia-Bueno et al., 2008; Joergensen et al., 2011). Moreover, high CRH levels stimulate the production of pro-inflammatory cytokines, thus further increasing oxidative stress (Wang et al., 2003). Importantly, increased stress levels and persistent negative emotions (e.g., as experienced during depression or life adversities), have been linked directly to accelerated biological aging through telomere shortening, DNA replication errors, and cellular damage (Lin et al., 2012; Price et al., 2013; Revesz et al., 2014; Blackburn et al., 2015; Zahran et al., 2015) or increased NF-κB activity (Irwin and Cole, 2011; Cole, 2014; Bower and Irwin, 2016). Furthermore, it has been shown that stress, anxiety, and depression are associated with reduced neuroplasticity and neurogenesis (Mcewen, 2001; Hanson et al., 2011; Liston and Gan, 2011; Besnard and Sahay, 2016), limiting the brain's ability to effectively adapt to new challenges. Finally, increased cortisol levels have been reported to be particularly detrimental in some brain regions (Bentson et al., 1978; Starkman et al., 1992, 1999; Knoops et al., 2010). For example, increased cortisol levels have been found to reduce the volumes of the hippocampus in rodents (Watanabe et al., 1992), primates (Uno et al., 1989), as well as humans (Bentson et al., 1978; Starkman et al., 1992, 1999; Knoops et al., 2010), suggesting a close link between stress, cortisol levels, and loss of brain tissue. Thus, while the stress response is protective in relation to transient threats, its chronic upregulation is detrimental and leads to accelerated (brain) aging.

KEY CONCEPT 3 **Glucocorticoids**

Glucocorticoid receptors are located inside the cells and, when activated, become transcription factors that change its functioning. On short term, glucocorticoids lead to multiple adaptations throughout the organism that are extremely beneficial during a stress response. However, when increased over a long time span, these changes can have detrimental effects.

### Macroscopic brain anatomy

A well-documented effect of aging is a progressive **decline in brain volume** commencing in our early twenties to mid-thirties. While it is not entirely clear how this volume loss is reflected at the microscopic level, significant contributors to tissue loss include decreases in neuron, glia, and spine numbers, reductions in interstitial space (neuropil), shrinkages of dendritic trees, and demyelination (Shen et al., 2008; Glorioso and Sibille, 2011). Importantly, age-related volume changes differ between tissue types and brain regions with different brain structures showing different trajectories. For total brain volume this decline is estimated to range from 0.2% per year in early to mid-adulthood to 0.6% per year in the sixties and seventies (Hedman et al., 2012; Tabatabaei-Jafari et al., 2015). In contrast, the hippocampus, reaches its maximum volume in the mid-twenties and then starts declining at a rate of 0.38% per year until the mid-fifties. The shrinkage rate then increases to 0.98% per year and peaks at 1.12% in the early seventies (Fraser et al., 2015). As for white matter, the age-related volume decrease has been reported to begin later than for gray matter, and maturation processes (e.g., fiber myelination) continue well into the thirties and possibly beyond (Westlye et al., 2010). Despite this continuous decline in brain tissue over time, the brain retains some ability to change and adapt to new demands across the lifespan (Greenwood and Parasuraman, 2010; Valkanova et al., 2014). Indeed, increases in local brain volume have been described in regions involved in the acquisition and performance of new tasks over time frames from several weeks to years, and importantly such positive changes were evident in young adults as well as in the elderly (Draganski et al., 2004, 2006; Boyke et al., 2008; Woollett and Maguire, 2011). This **neuroplasticity** may allow, at least to some extent, to compensate for age-related volume loss by consolidating and optimizing resources (for review see Greenwood and Parasuraman, 2010). Similarly, factors such as educational attainment have been proposed to stimulate neuroplasticity and neurotrophic mechanisms, thereby enhancing brain and cognitive reserves, which may ultimately manifest as more age-resilient brains (Stern, 2012).

KEY CONCEPT 4 **Decline in brain volume**

Probably one of the best established effects of aging in brain research is a progressive loss of brain volume. While volume loss may be compensated to some degree via neuroplastic adaptation, it may reach a threshold below which full compensation becomes impossible. A relative preservation of volume might therefore be protective or delay the onset of functional decline.

KEY CONCEPT 5 **Neuroplasticity**

The brain has the ability to adapt in response to the existence (or absence) of demand. Neuroplasticity could be observed in local volume increases as a response to the learning of new tasks. Neuroplastic changes on a macroscopic level can be observed within weeks to years and can be assumed to be a continuous and fundamental process.

## Effects and possible mechanisms of meditation

While there is only limited research investigating meditation and biological processes implicated in brain aging and neurodegeneration, it hints at plausible pathways through which meditative practice may exert beneficial effects on (brain) aging. In the next sections, we will review this emerging field of research suggesting an association between meditation and the aforementioned aspects and processes as pertaining to normal aging.

### Effects of meditation on telomeres

As described above (see Section Telomeres), the enzyme telomerase contributes to repairing accumulated DNA damage and extending shortened telomeres. Interestingly, recent studies have indicated that meditation may increase telomerase activity (Jacobs et al., 2011; Lavretsky et al., 2013; Schutte and Malouff, 2014) and telomere length (Hoge et al., 2013; Alda et al., 2016). While telomere *length* has only been measured cross-sectionally, telomerase *activity* has been investigated longitudinally. Altogether, these studies' outcomes are consistent with the notion that meditation is protective against cellular aging. However, some caution is warranted. Telomere length and telomerase activity are measured in leukocytes from blood draws, and therefore these biomarkers may only reflect conditions (and possible meditation-induced effects) in peripheral blood cells, rather than in neurons and more broadly brain tissue. In fact, the role of telomerase in cells of the central nervous system remains obscure. More specifically, even although there is some preliminary evidence that a subunit of telomerase is also expressed in neurons after brain injury, it remains unclear if telomerase is expressed under normal conditions and with what result (Mattson et al., 2002; Zhang et al., 2007; Gonzalez-Giraldo et al., 2016). It should also be noted that while the critical role of telomere attrition is well-established in cell aging, its specific effect on the brain is not entirely clear (Allsopp et al., 1995; Nakamura et al., 2007; Zhang et al., 2010; Eitan et al., 2014). Nevertheless, shorter leukocyte telomere length has been reported to predict dementia, cognitive decline, and mortality, suggesting at least some link between telomere length and brain aging (Eitan et al., 2014). Moreover, as telomeres are particularly sensitive to DNA damage (see Section Telomeres), the positive findings in meditators (i.e., longer telomeres, stronger telomerase activity) may point to less inflammatory signaling (see Section Inflammation), less stress (see Section Stress Regulation), and as a result more functionally intact cells, at least within the blood stream. Unfortunately, more comprehensive investigations exploring these links in controlled prospective intervention studies, optimally over an extended time frame, are entirely missing.

### Effects of meditation on inflammation

Recent longitudinal studies have revealed that meditation reduces NF-κB activity (Creswell et al., 2012; Bhasin et al., 2013; Black et al., 2013; Bower et al., 2015; Rao et al., 2015; Bower and Irwin, 2016) as well as circulating markers of inflammation, such as C reactive protein and interleukin (IL)-6 (Creswell et al., 2012, 2016; Lengacher et al., 2012; Malarkey et al., 2013). Moreover, the reduced NF-κB activity was accompanied by a meditation-induced increased activity of the anti-inflammatory glucocorticoid receptor, which suggests a higher sensitivity to anti-inflammatory glucocorticoids (Bower et al., 2015; Bower and Irwin, 2016). Overall, these results point to a reduced pro-inflammatory state in active meditation practitioners. However, as for telomerase activity and telomere length (as discussed in Section Effects of Meditation on Telomeres), markers of inflammation are measured in peripheral leukocytes, rather than in neurons of the central nervous system; and although the role of NF-κB in brain cells is better established than for telomeric measures, the interpretation of these findings remains tentative. That is, on the one hand, NF-κB within glia cells was found to exert a pro-inflammatory activity and the potential to cause neuronal degeneration. On the other hand, and outside the realm of inflammation, NF-κB has also been established as an important factor for neuronal development, plasticity, repair, and survival (Mattson, 2005; Mattson and Meffert, 2006; Camandola and Mattson, 2007; Boersma et al., 2011). Thus, a simple extrapolation from findings in peripheral leukocytes to all cells and circumstances may be too simplistic, especially as it is well-known that single factors can result in different effects depending on the circumstances. Overall, however, meditation-induced reduction in NF-κB activity might be beneficial, as inflammatory activity observed in peripheral immune cells (e.g., leucocytes) may be correlated (at least to some degree) with the activity of immune cells in the brain (e.g., microglia). Overall, these reductions of NF-κB activity may potentially reflect a delay of the smoldering pro-inflammatory phenotype “inflammaging” and its detrimental effects (see Section Inflammation), although this latter hypothesis remains mere conjecture until it can be tested directly. Along these same lines, a meditation-induced increase in the activity of the glucocorticoid receptor (Bower et al., 2015; Bower and Irwin, 2016) is likely to increase the anti-inflammatory properties of cortisol. This, in turn, may help reduce cortisol levels overall (because the higher glucocorticoid receptor sensitivity will relay the same anti-inflammatory effects at lower doses of circulating cortisol). As may be inferred from the discussion of the detrimental effects of cortisol (see Section Stress Regulation), decreased cortisol levels are likely to contribute to lower rates of brain shrinkage, and particularly so in the hippocampus. So, altogether, the observed effects of meditation on inflammation processes may have beneficial effects on brain aging. However, comprehensive controlled trials to substantiate these effects are still missing.

### Effects of meditation on stress regulation

Several studies suggest that meditation fosters adaptive emotion regulation and emotional well-being (Moynihan et al., 2013; Grecucci et al., 2015; Zeng et al., 2015), while also reducing stress and cortisol levels (Chiesa and Serretti, 2009; Koike and Cardoso, 2014; Ray et al., 2014; Sharma and Rush, 2014; Turan et al., 2015). Such effects were not only observed cross-sectionally when comparing meditators with non-meditators but also longitudinally in randomized controlled trials exploring the effects of meditation. These findings may reflect improved voluntary regulation of mental activity, shifting focus and attention, as well as reduction in rumination (Holzel et al., 2011; Wolkin, 2015). As discussed above (see Section Stress Regulation), stress reduction may play a central part in the modulating effects of meditation on brain aging, especially as chronically increased cortisol levels have been associated with increased ROS, NF-κB activity, and brain volume loss, as well as reduced telomere length and neuroplastic capacity (see Section Inflammation and Stress Regulation). Thus, while stress is not a mechanism of aging *per se*, it can directly affect a multitude of aging processes. Unfortunately, a comprehensive assessment of the effects of meditation on perceived stress, objective biomarkers of stress (including cortisol levels etc.), changes in telomere length, NF-κB activity, and brain tissue over an extended time is not yet available. This severely limits the level of inference that can be drawn at this point. More specifically, while links and interactions between meditation, stress regulation, and mechanisms of aging appear likely, they have not been assessed directly and remain a hypothesis that awaits to be tested in future studies.

### Effects of meditation on macroscopic brain anatomy

A limited number of cross-sectional neuroimaging studies has investigated the possible modulating impact of meditation on age-related changes in the macrostructure of the brain (Luders, 2014; Luders and Cherbuin, 2016). These investigations revealed less pronounced negative correlations between chronological age and various cerebral measures (e.g., local and global gray matter volumes or indicators of white matter fiber integrity) in meditators than in controls (Lazar et al., 2005; Pagnoni and Cekic, 2007; Luders et al., 2011, 2015; Kurth et al., 2015; Laneri et al., 2016). Moreover, when estimating the age of brains using automated pattern recognition, the brains of meditators appeared to be several years younger than the brains of age-matched controls (Luders et al., 2016). These findings seem to suggest that meditation may slow down age-related brain degeneration. However, the cross-sectional nature of these studies precludes an inference on causality. That is, it remains unclear whether meditation induced the observed differences, whether the differences existed already before people started meditating, whether there is interplay between the two, or whether the differences are due to more complex interactions with other factors. Assuming the observed differences are the result of meditation, a combination of processes is likely to be implicated (Kurth et al., 2015; Luders et al., 2015; Luders and Cherbuin, 2016): For example, through a variety of mechanisms, such as upregulating telomerase activity, down-regulating pro-inflammatory processes, and decreasing the effects of chronic stress (see Sections Effects of Meditation on Telomeres, Effects of Meditation on Inflammation, and Effects of Meditation on Stress Regulation), meditation may prevent or at least reduce the normal age-related brain decline by averting neurotoxic effects on the brain. However, these effects might even go beyond the mere prevention of tissue loss and actually present as tissue gain, at least in the short term. For example, it has been reported that loss of hippocampal tissue originally induced by high cortisol levels was restored when cortisol levels were reduced (Starkman et al., 1999). Similarly, relative tissue increases may occur due to anabolic training effects, similar to the neuroplasticity observed in imaging studies (see Section Macroscopic Brain Anatomy). In fact, several longitudinal studies revealed changes in local brain tissue after 4–8 weeks of intense meditation and, overall, these changes presented as increases (Fox et al., 2014). Nevertheless, some tissue decreases were also evident in brain regions known to modulate stress and fear responses and/or when effects of meditation were examined in pathological states (Holzel et al., 2010; Kurth et al., 2014). Although a time frame of several weeks is likely too short to draw sensible conclusions with respect to the impact of meditation in the framework of aging, the existing short-term longitudinal studies already demonstrate meditation-induced neuroplastic changes in brain structure. Controlled long-term longitudinal studies might reveal even more pronounced changes, especially if conducted over several years. Ideally, such trials will also obtain detailed measures of stress, inflammation, and telomere attributes to facilitate the interpretability of findings.

## Summary and outlook

Aging is happening to each and all of us every single day. It is an ongoing process regardless of our birth dates, but the obvious sequels become most evident late in life. Aging is complex and our understanding of it still resembles an incomplete puzzle. However, we know that throughout the life span, there are multiple factors that may influence the dynamics of aging, and meditation may be one of them. Without a doubt, the accumulating scientific evidence is very encouraging, especially given that meditation is relatively easy to integrate in everyone's every-day life. Nevertheless, numerous open questions exist for diverse aspects, ranging from missing answers on the biological level, over the specifics of an effective (personalized) meditation intervention, to the general question of causality. Moreover, preliminary evidence for possible age-defying effects of meditation mostly stems from cross-sectional studies and/or from using indirect markers associated with aging. In contrast, controlled longitudinal studies between meditation and diminished brain aging are still missing. Admittedly, since aging is a rather slow and dynamic process, such prospective trials constitute challenging long-term endeavors but they seem necessary eventually to prove a causal relationship. In conclusion, before constructing effective prevention and/or intervention plans leveraging the potential of meditation to affect multiple pathways involved in (brain) aging, future research is clearly necessary to continue collecting still missing pieces and painting a more complete picture.

## Author contribution

All authors FK, NC, EL contributed equally in writing and revising the manuscript.

### Conflict of interest statement

The authors declare that the research was conducted in the absence of any commercial or financial relationships that could be construed as a potential conflict of interest.
